# Myopia risk behaviour related to the COVID-19 lockdown in Europe: The generation R study

**DOI:** 10.1111/opo.13100

**Published:** 2023-02-11

**Authors:** Sander C. M. Kneepkens, Jimmy de Vlieger, J. Willem L. Tideman, Clair A. Enthoven, Jan Roelof Polling, Caroline C. W. Klaver

**Affiliations:** 1 Department of Ophthalmology, Erasmus Medical Center, Rotterdam, The Netherlands; 2 The Generation R Study Group, Erasmus Medical Center, Rotterdam, The Netherlands; 3 Martini Hospital, Groningen, The Netherlands; 4 Department of Psychology, Education and Child Studies, Erasmus University, Rotterdam, The Netherlands; 5 Department of Child and Adolescent Psychiatry, Erasmus Medical Center, Rotterdam, The Netherlands; 6 University of Applied Sciences, Utrecht, The Netherlands; 7 Radboud University Medical Center, Nijmegen, The Netherlands; 8 Institute of Molecular and Clinical Ophthalmology, Basel, Switzerland

**Keywords:** axial length, COVID-19, myopia, quarantine, refractive error, spherical equivalent refraction

## Abstract

**Purpose:**

To battle the spreading of the COVID-19 virus, nationwide lockdowns were implemented during 2020 and 2021. Reports from China revealed that their strict home confinements led to an increase in myopia incidence. The Netherlands implemented a more lenient lockdown, which allowed children to go outside. We evaluated the association between COVID-19 restrictions, myopia risk behaviour and myopia progression in Dutch teenagers.

**Method:**

A total of 1101 participants (mean age 16.3 ± 3.65 yrs) completed questionnaires about their activities before, during and after lockdown (March–October 2020). We used a repeated-measures ANOVA to compare time use between these time periods. Ocular measurements were acquired before the COVID-19 pandemic when participants were 13 years old; only 242 participants had ocular measurements at 18 years of age at the time of this analysis. Linear regression analyses were used to evaluate the association between lifestyle factors and myopia progression.

**Results:**

Children were on average 16.2 (1.03) years of age during lockdown. Total nearwork increased from 8.11 h/day to 11.79 h/day, and remained higher after lockdown at 9.46 h/day (*p* < 0.001). Non-educational nearwork increased by 2.22 h/day (+49%) during lockdown and was associated with faster axial length progression (B 0.002 mm/h/year; SE 0.001 *p* = 0.03). Before and during lockdown, the mean time spent outdoors was similar (1.78 h/day and 1.80 h/day, respectively). After lockdown, time spent outdoors decreased to 1.56 h/day (*p* < 0.001).

**Conclusion:**

The Dutch lockdown significantly increased digitised nearwork in adolescents but did not affect outdoor exposure. The changes in time spent performing nearwork remained after the lockdown measures had ended. We expect that the COVID-19 pandemic may lead to an increase in myopia prevalence and progression in European children.

**Supplementary Information:**

The online version of this article (doi:10.1111/opo.13100) contains supplementary material, which is available to authorized users.

## Key Points

- This study investigated whether the public health measures taken to counteract the COVID-19 pandemic in Europe were associated with an increase in myopia progression in teenagers.
- The findings revealed that the Dutch COVID-19 lockdown pushed teenagers towards a myopic risk profile leading to an increase in myopia progression, a push that was sustained even after the lockdown ended.
- The COVID-19-related lockdown created ‘a new normal’ for teenage behaviour in Europe, which adds to the already increasing prevalence of myopia.

## INTRODUCTION

Public health pandemic control measures designed to prevent the spread of the novel coronavirus (SARS-CoV-2) disease known as COVID-19 resulted in numerous changes to daily living. Restrictions varied from home confinement, where time outdoors was forbidden to more lenient lockdowns, which allowed time outdoors. Europe has seen the highest number of COVID-19 cases (204 million), followed by the Americas (150 million) and South-East Asia (57 million).^1^ Although lockdown measures were highly effective in containing the virus, they also showed a serious negative effect on mental, physical and eye health, especially in children and young adults.^2–4^

The global myopia boom began well before the COVID-19 pandemic. Currently, almost 50% of European young adults and 80%–90% of Chinese university students appear to be myopic.^5–7^ It is expected that by 2050, 43%–56% of the world population will have become myopic, and 6%–19% will have become highly myopic.^8^ Myopia can result in irreversible visual impairment or blindness due to retinal complications such as myopic macular degeneration, retinal detachment and glaucoma.^9,10^ Risk factors for myopia are little time spent outdoors and long hours focusing at a short distance such as looking at electronic screens.^11–13^ Both of these factors were exacerbated by home confinement.^14–17^ Chinese studies reported that myopia prevalence increased during the COVID-19 pandemic. A recent meta-analysis found an annual myopia progression of 0.6 D measured by cycloplegic refraction in several combined studies.^18^

Current evidence of the increase in myopia during the COVID-19 pandemic stems from Chinese cohort studies.^15,19^ Lockdown measures in Asia involved tight restrictions and total home confinement, whereas more lenient policies facilitated outdoor time in the Netherlands and many other European countries.^20^ Currently, no studies have reported on myopia progression due to COVID-19 pandemic–related public health measurements in European cohorts. Whether a less stringent lockdown had the same effect on myopia as complete home confinement is unknown. In this study, we aimed to investigate the association between COVID-19 restrictions in the Netherlands, and myopic risk profile and myopia progression in young adolescents, combining data from questionnaires and measurements of refractive errors and axial lengths (ALs).

## METHODS

### Study design and participants

This study was embedded into the Generation R Study, a large ongoing population-based prospective birth cohort of 9778 pregnant women and their children who were born between April 2002 and January 2006 in Rotterdam, the Netherlands. Details of the methodology have been described elsewhere.^21,22^ Participants were invited at 5, 9 and 13 years of age for examinations, including eye measurements, and received extensive questionnaires about lifestyle, socioeconomic status and health. The follow-up round at age 18 is currently taking place; examinations started in October 2020 but were interrupted by lockdowns due to COVID-19. This round is expected to be completed by January 2024. The study protocol was approved by the Medical Ethical Committee of the Erasmus Medical Center, Rotterdam (MEC 217.595/2002/20) and written informed consent was obtained from all parents and participants.

### Eye measurements

Automated cycloplegic refractive error data were available from the third round at 13 years of age, and partly available from the ongoing round at 18 years of age. At 13 years of age, cycloplegia was achieved with two to three drops of cyclopentolate 1% instilled in both eyes. At 18 years of age, this was achieved by two to three drops of tropicamide (1%) instilled into the right eye only (OD). Myopia was defined as spherical equivalent refraction (SER) ≤ −0.5 dioptre in the right eye (*N* = 117). Ocular biometry was measured with the Zeiss IOL-master 700 (Zeiss.com, *N* = 242). For AL, five measurements per eye were averaged. Additionally, the axial length corneal curvature ratio (ALCR) and mean axial elongation (Δmm/year) were calculated.

### COVID-19 questionnaire

**FIGURE 1:**
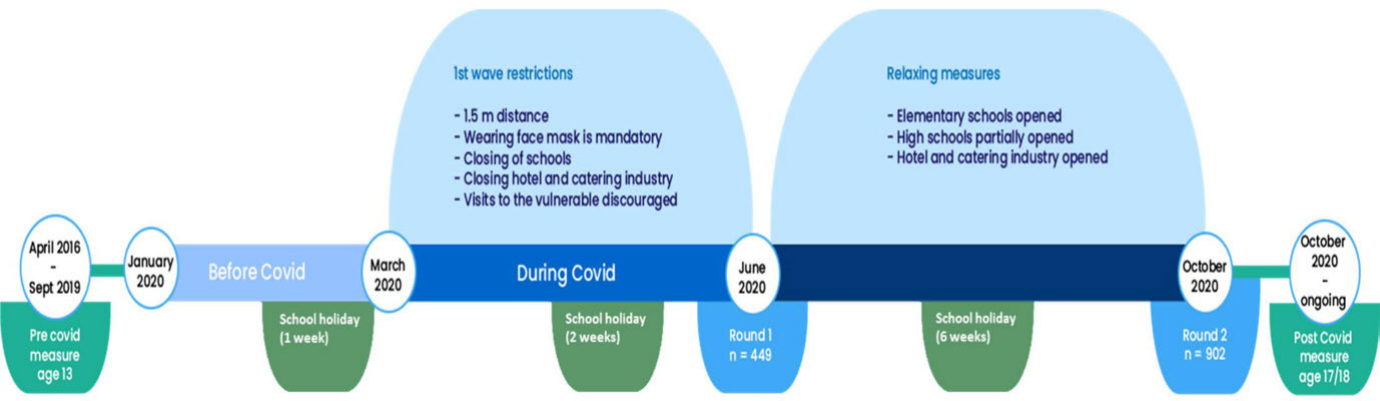
Timeline of lockdown and restrictions to prevent the spread of COVID-19 in the Netherlands in 2020.

COVID-19 questionnaires were sent to all participants in the Generation R Study in June and October 2020. In the Netherlands, lockdown was enforced from March to June 2020 (Figure 1). The Dutch government implemented a ‘partial lockdown’ whereby schools were closed and bars and restaurants were only open for take away, but there was no home confinement.^23^

The first questionnaire gathered data from two time points, that is before and during lockdown. The second questionnaire questioned behaviour during a third time point, that is after lockdown.

With respect to activities, participants answered questions about how much time they spent before, during and after the lockdown on non-educational nearwork activities (e.g., watching programmes or playing games on their phone or TV or reading a book for fun), educational nearwork activities (doing homework online or offline), time spent online (on the internet) and time spent outdoors. Response options were divided into categories: ‘0-30 minutes each day, 30-60 minutes each day, 1-2 hours each day, 2-4 hours each day and 4-6 hours each day’. With respect to covariates, educational level was categorised into lower secondary, upper secondary, post-secondary non-tertiary education and tertiary education according to the UNESCO classification.^24^ Sex registered at birth was categorised into male and female. We excluded participants with answers that were outside the normal range (e.g. total time/day >1440 min or >24 h).

### Statistical analysis

Participants were included when data were available for all time points: before, during and after lockdown. First, we divided nearwork into separate categories. Non-educational nearwork was calculated by adding up any non-school related nearwork; educational nearwork was calculated by adding up all school-related nearwork. In addition, we categorised screen time by medium. Smartphone use was calculated as the sum of smartphone use for TV programmes, games and social media. Remote device use was the sum of computer, TV or projector use for TV programmes and games. Undefined device use was the sum of screen time where no specific device was defined in the question. Next, these categories (dependent variable) were compared before, during and after lockdown (independent variable) using a repeated-measures ANOVA; post-hoc pairwise comparisons were made using Bonferroni correction.

A subset (*N* = 242) of the data was used to determine the association between time spent on nearwork activities and myopia progression using linear regression models stratified by time point (before, during or after lockdown). Multiple models were used; as outcome, we used either AL elongation (mm/year) or SER change (D/year). As predictor, we used either time spent on a smartphone, remote devices, non-educational nearwork or educational nearwork. All models were adjusted for potential confounders such as age, gender and outdoor exposure. In addition, conditional regression analysis was used to identify whether a change in time spent on non-educational nearwork and remote device during a particular period (pre-, during or post-lockdown) was most associated with axial elongation by adjusting for previous time use.^13,25^ Three conditional time points were calculated by saving the standardised residuals of the regression analyses: time spent before lockdown, time spent during lockdown adjusted for time spent pre-lockdown, and time spent post-lockdown adjusted for time use pre- and during lockdown (formula can be found in Table S2). This resulted in an estimate of increase per time interval, independent of the previous time spent on nearwork and remote devices. Next, the conditional analyses were performed with ocular biometry as the dependent variable and the standardised residuals of each time point as the independent variable. This resulted in an effect per time interval.^26^ Cycloplegic refractive error and AL were compared pre- and post-lockdown by repeated-measures ANOVA. Since we used complete case analysis, we assumed missingness completely at random.

## RESULTS

### Subject characteristics

The 760 children with complete data at all three timepoints entered the analyses; their demographics and general characteristics are provided in Table 1. The mean age was 16.2 years with 42% male and 80% of European ethnicity. The participants were relatively highly educated with 64% in upper secondary education, whereas the average in the Netherlands is 55%.^27^ As the fourth round of Generation R was still ongoing at the time of this analysis, post-COVID lockdown eye measurements were available only in a subset of 242 participants.

**TABLE 1:** Demographics of the study population

Demographics	Total study population	Pre-lockdown eye examination	Post-lockdown eye examination
Age in years, mean (SD)	16.2 (1.03)	13.63 (0.29)	18.38 (0.43)
Sex, % male (*N*)	42.4 (322)	37.2 (89)	37.2 (89)
European ethnicity, % (*N*)	80.5 (612)	81.3 (196)	81.3 (196)
Education			
Lower secondary, % (*N*)	10.4 (79)	-	4.5 (11)
Post-secondary non tertiary, % (*N*)	11.1 (84)	-	16.1 (39)
Upper secondary, % (*N*)	68.4 (520)	-	59.1 (143)
Tertiary, % (*N*)	7.4 (56)	-	15.7 (38)
Subjects in analysis, *N*	760	242	242

### Time spent outdoors and online on the internet

**FIGURE 2:**
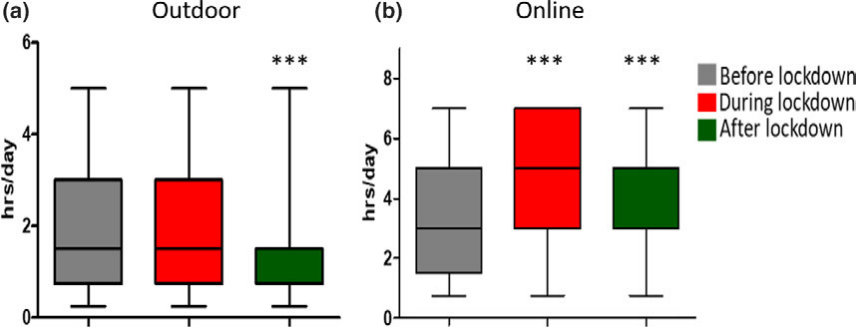
Median time spent outdoors (a) and online (b) in hours per day before, during and after lockdown. Differences were significant compared with before lockdown: ***p < 0.001.

The questionnaires before and during lockdown were sent out in spring and summer, respectively. The mean time spent outdoors during these periods was 1.78 h/day and 1.80 h/day. The questionnaire after lockdown was completed during the autumn season, between 1 September and 30 November. Outdoor exposure during this period was 1.56 h/day (*p* < 0.001 compared with before lockdown; see Figure 2a). The average time spent online was 3.29 h/day before lockdown, increasing to 4.80 h/day (*p* < 0.001) during lockdown. This activity remained significantly higher after the lockdown (3.91 h/day, *p* < 0.001; Figure 2b). No significant difference was found between myopic and non-myopic participants.

### Nearwork

**FIGURE 3:**
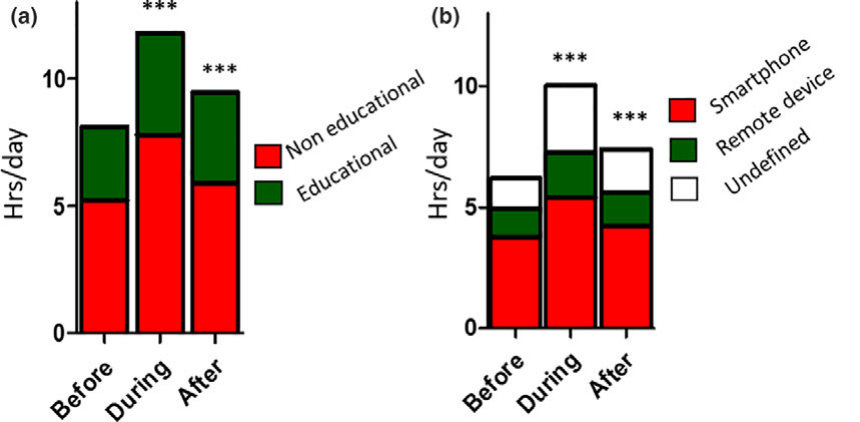
Time spent on nearwork (a) and screen time (b) in hours per day before, during and after lockdown, stratified into non-educational and educational nearwork, as well as into smartphone, remote devices and undefined devices. Differences were compared with the before lockdown values for both total and stratified nearwork and screen time: *** = *p* < 0.0001.

Time spent on social media, watching programmes, playing games and reading for leisure were analysed together as non-educational nearwork. Conversely, time spent on schoolwork both on and offline was considered educational nearwork. Total time spent on nearwork before, during and after lockdown was 8.11 h/day, 11.79 h/day and 9.46 h/day, respectively. These represented an increase of 45% and 17% compared with before lockdown (both *p* < 0.001). Educational nearwork increased from 2.89 h/day before lockdown to 4.02 h/day during lockdown, an increase of 39% (*p* < 0.001). This reduced to 3.57 h/day after lockdown, which was still 23% higher than before lockdown (*p* < 0.001; Figure 3). The average time spent on non-educational nearwork was higher than on educational nearwork but showed the same pattern, with values before, during and after lockdown of 5.22 h/day, 7.77 h/day (49% increase) and 5.89 h/day (13% increase), respectively (both increases *p* < 0.001; Figure 3). No significant difference was found between myopic and non-myopic participants.

### Screen time

Time spent on games, videos, social media and online schoolwork was summed up as screen time. Screen time before, during and after lockdown was 6.19, 10.02 and 7.39 h/day, respectively. The latter two were 61% and 19% higher than before lockdown (both *p* < 0.001; Figure 3b). We stratified screen time into smartphone, remote devices and undefined device. Average times spent on the smartphone before, during and after lockdown were 3.76, 5.40 and 4.25 h/day, respectively, corresponding to increases of 43% and 13%, respectively (both *p* < 0.0001; Figure 3b). The equivalent times for remote devices were 1.19, 1.85 and 1.37 h/day, representing an increase of 56% (*p* < 0.0001) and 12% (*p* < 0.001; Figure 3b). No significant difference was found between myopic and non-myopic participants.

**FIGURE 4:**
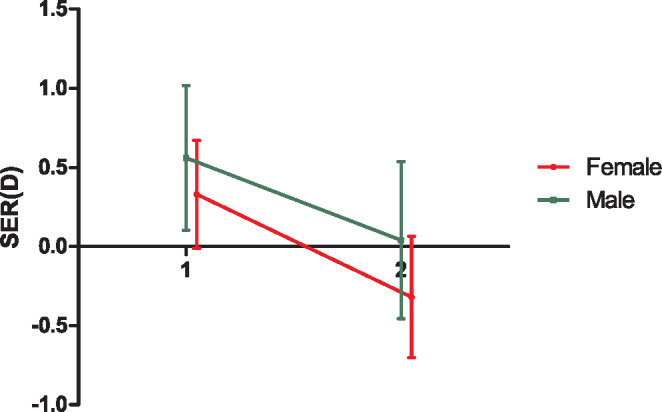
Spherical equivalent refractive error (SER) measured at timepoint 1 (age 13 years, pre-lockdown) and timepoint 2 (age 18 years post-lockdown) stratified for male (green) and female (red) subjects. Error bars indicate 95% CI.

### Eye measurements

Although all participants had undergone eye examinations during previous rounds of Generation R, only 89 boys and 154 girls had been tested in the currently ongoing round. Hence, measurements of ocular biometry were available for these individuals both pre- and post-COVID (Table 2). Cycloplegic measurements of refractive error at both time points were available for 45 boys and 72 girls. The mean axial elongation during the 13- to 18-year age period was similar for both sexes (boys: 0.05 ± 0.03 mm/year; girls: 0.05 ± 0.05 mm/year). Average SER had shifted towards plano for boys (+0.63 D to +0.05 D) and had become myopic for girls (+0.33 D to −0.32D; Figure 4). Myopia prevalence increased by 2.8% for boys to a prevalence of 20.6%; for girls, it increased by 11.1% to a prevalence of 33.3% (Table 2).

**TABLE 2:** Eye measurements collected pre- (age 13) and post-COVID (age 18) and divided by sex

	Boys (*N* = 89)		Girls (*N* = 154)	
Ocular biometry	Pre-lockdown	Post-lockdown	Pre-lockdown	Post-lockdown
Age in years, mean (SD)	13.58 (0.24)	18.40 (0.41)	13.66 (0.31)	18.35 (0.46)
Axial length, mm (SD)	23.52 (0.85)	23.75 (0.91)	23.39 (0.88)	23.63 (0.98)
Axial elongation, mm/year (SD/range)	-	0.05 (0.03/−0.03 to 0.15)	-	0.05 (0.05/−0.04 to 0.23)
Axial length to corneal radius ratio (SD)	3.01 (0.09)	3.03 (0.10)	3.01 (0.10)	3.04 (0.11)
Cycloplegic refraction^a^				
Prevalence of myopia, % (*N*)	17.8 (8)	20.6 (9)	22.2 (16)	33.3 (24)

^a^ Available in a subset of participants (male *N* = 45; female *N* = 72).

**FIGURE 5:**
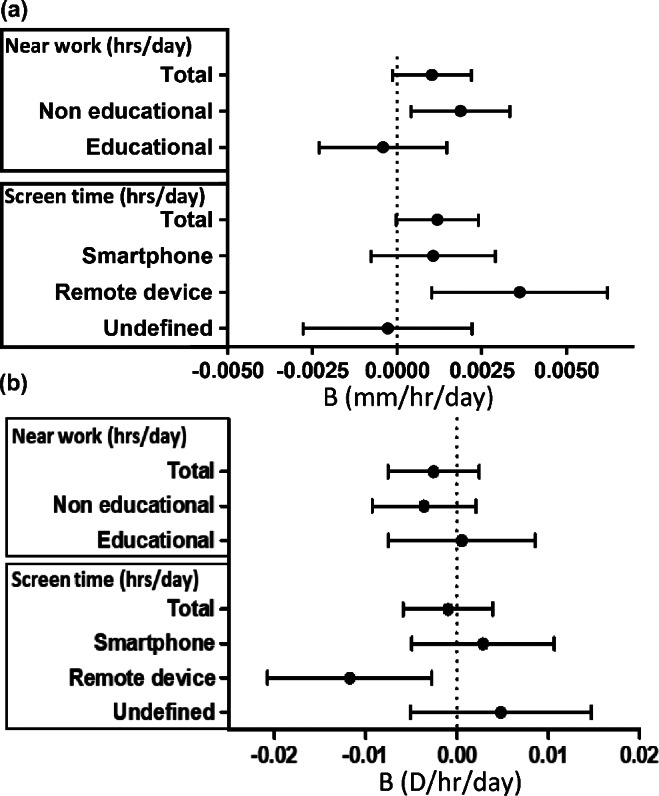
Beta estimates and 95% confidence intervals for a linear regression model of (a) yearly axial elongation (mm/year) and (b) spherical equivalent refractive error change (D/year) for nearwork (h/day) and screen time (h/day) during lockdown. D, dioptres.

The association between the nearwork performed around the lockdown period and annual axial elongation was investigated using a linear regression model corrected for age and gender. More time spent on non-educational nearwork during lockdown was associated with higher annual axial elongation (B 0.003 mm/h/day; CI: 0.0005–0.0050; *p* = 0.02). Time spent on remote devices during lockdown was also significantly associated with axial elongation (B 0.003 mm/h/day; CI: 0.001–0.006; *p* = 0.007; Figure 5a). In addition, we performed these analyses using ΔSER/yr as outcome and found a significant decrease in SER for remote device use before (B −0.015 D/h/day; CI: −0.028 to −0.001; *p* = 0.03) and during lockdown (B −0.012 D/h/day; CI: −0.21 to −0.003; *p* = 0.009; Figure 5b). Other associations were in the same direction but did not reach statistical significance. Conditional regression analysis was used to identify the most important time period by adjusting for previous time spent on non-educational nearwork and remote device use. The strongest association with axial elongation was found during lockdown (B 0.009 mm/h/day; CI: 0.002–0.016; *p* = 0.02). Conditional regression analysis for remote device use also showed the strongest association with both axial elongation and ΔSER/year during lockdown (B 0.041 mm/h/day; CI: 0.040–0.042; *p* = 0.03; B 0.120 D/h/day; CI: 0.117–0.124; *p* = 0.001).

## DISCUSSION

This study showed that the COVID-19 lockdown in the Netherlands aggravated a myopic risk profile and may have accelerated myopia progression. Participants spent more time on screens and nearwork during lockdown, and their use of remote devices and smartphones remained higher even after the lockdown had ended. Additionally, more time spent on nearwork during lockdown was associated with AL elongation and a myopic shift in the SER, even without a reduction in outdoor exposure. Moreover, conditional regression analysis showed the strongest association during lockdown.

Lockdown measures varied greatly across the globe. In the Netherlands, we used a so-called ‘intelligent’ or partial lockdown. Schools were closed and bars and restaurants were only open for takeaway, but there was no home confinement.^23^ This may explain why we did not find a significant difference in outdoor exposure during lockdown compared with beforehand. The first relaxation of restrictions in the Netherlands began in June 2020. However, the after-lockdown period that participants were asked about in the present study was specified as the first 2 weeks of October. Autumn begins on 23 September in the Netherlands, and this seasonal change likely explains the decreased time spent outdoors after lockdown. In the earlier round performed at 13 years of age, a similar pattern was observed for time spent outdoors (2.6 h/day in the summer vs. 2.2 h/day in the autumn; *p* < 0.001). For nearwork, this earlier difference approached significance but was much smaller than our observed increase (summer 3.4 h/day vs. autumn 3.7 h/day; *p* = 0.06; Figure S1A,B). In addition, daylight hours vary greatly during the pre-, during and post-lockdown periods; in Rotterdam, these are 11 h, 15–16 h and 11 h, respectively, with latitude and longitude coordinates of 51.927 and 4.462°.^28^ At many other places around the world, governments imposed strict home confinements for the entire family during lockdown. For example, Chinese and Argentinian studies found a significant decrease in time spent outdoors during lockdown.^15,16,18^ In China, outdoor activities were forbidden and strictly enforced so that children experienced zero minutes per day outdoors.^16^ Outdoor exposure is widely accepted as an important protective factor for myopia progression,^29^ and the more lenient restrictions may have safeguarded Dutch teenagers from more rapid progression. For example, the myopic progression observed here was 0.60D over 3 years, while in a recent meta-analysis of studies from Turkey, Argentina and China, this degree of myopia progression was reached within 1 year.^18^

This is the first European COVID-19 study that observed a significant relationship between non-educational nearwork and myopia progression. Globally, daily screen time has increased by approximately two hours between 2002 and 2010,^30^ while during the COVID-19 pandemic an immediate increase of approximately one hour was observed.^31,32^ Studies on other continents also reported an increase in nearwork risk behaviour during lockdown,^14,16,33^ which remained higher afterwards. Time on screens increased by 59–62 min a day in a Dutch cohort and by as much as 30 hours a week in a Chinese cohort.^31^ Our findings confirm these observations and suggest that the pandemic will lead to an even higher prevalence of myopia than was predicted prior to COVID-19.^8^

Self-reported data were used to quantify time use, and each question had six response options. This could have led to self-report bias, a limitation that may in turn have caused an underestimation of the time spent on tasks, as people tend to report more favourable answers when asked to describe their behaviour.^34^ Likewise, this may have led to an overestimation of outdoor exposure.

An important strength of this study is the longitudinally collected data. The current round at 18 years of age is still ongoing; therefore, only a subsample of our total participants have undergone eye measurements post-COVID. Nevertheless, this limited sample was larger than that of other myopia studies from the Western world.^18,33^ Another strength is that our cohort is a good representation of European society. It is urban and ethnically very diverse, with participants from both low and high socioeconomic classes. The cohort was slightly higher educated than the average young Dutch population (55% vs. 68.4%).^27^ Higher-educated children tend to spend less time on screens, and so this might cause an underestimation of the time spent on non-educational nearwork.^35^ In addition, pre-COVID measurements were obtained approximately 3 years before the post-COVID data. Therefore, the true effect of life style changes during lockdown was probably diluted by statistical noise, and only a small effect on myopia progression can be expected. Additionally, the responders to this questionnaire were compared with the entire cohort. This group was significantly higher educated (total cohort vs. questionnaire responders; *p* < 0.001), had a higher proportion of girls (42.4% for the total cohort vs. 49.1% for the questionnaire responders; *p* < 0.001) and a higher proportion of Europeans (72.8% for the total cohort vs. 80.5% for the questionnaire responders; *p* < 0.001).

## CONCLUSION

Non-educational nearwork during the COVID-19 pandemic may be associated with an increase in myopia prevalence and progression. The COVID-19 pandemic had a prolonged and potentially permanent effect on the behaviour of Dutch adolescents that may cause a sharp increase in myopia prevalence and progression in the near future. However, myopia progression was lower than in other countries, which may be related to more time spent outdoors. The study findings highlight the importance of acknowledging and countering myopic progression when a lockdown is imposed, as well as the need to enforce frequent breaks from nearwork and promote engagement in outdoor activities now that lockdowns are becoming less frequent.

## Supplementary Information

Supplementary file (DOCX 44.2 KB)
