# Supplementary material for: Myopia risk behaviour related to the COVID-19 lockdown in Europe: The generation R study
Source: Ophthalmic Physiol Opt. 2023 Feb 11;43(3):402–9. doi: 10.1111/opo.13100 (PMC12852137; doi:10.1111/opo.13100)
Supplement: Supplementary file 1 — Supplementary file (DOCX 44.2 KB) [file 44402_2023_4303012_MOESM1_ESM.docx]

 ​Supplementary table 1: Comparison of patient demographics between total cohort during last visit at age 13 and responders of the COVID questionnaires.

| **Demographics** ​ | Total Cohort (N=4102) | COVID questionnaires | P Value (X^2^) |  |  |  |
| --- | --- | --- | --- | --- | --- | --- |
| Age, mean (SD) | 13.53 (0.35) | 16.2(1.03) ​ |  |  |  |  |
| Sex, % male (N) ​ | 49.1(2016) | 42.4 (322)​ | p<0.0001 |  |  |  |
| European ethnicity,​ % (N) | 72.8 (2986) | 80.5 (612)​ | p<0.0001 |  |  |  |
| ***Education***​ |  | ​ | p<0.0001 |  |  |  |
| *Primary* | 1.6 (67) |  |  |  |  |  |
| Lower secondary​, % (N) | 31.0 (1273) | 10.4(79)​ |  |  |  |  |
| Post-secondary non tertiary, % (N)​ | - | 11.1 (84) |  |  |  |  |
| Upper secondary​, % (N) | 65.5 (2687) | 68.4 (520)​ |  |  |  |  |
| Tertiary​, % (N) | - | 7.4 (56) |  |  |  |  |
| Subjects in analysis, N ​ | 4102 | 760​ |  |  |  |  |
| ​ | ​ | ​ |  |  | ​ | ​ |

Supplementary table 2: Equation and interpretation of conditional regression analysis.

| **Model description** | **Equation** |
| --- | --- |
| Early model, regression analysis of time use before lockdown to outcome (Axial elongation&SER).…. ..……. | $Y=\alpha_{1}+\beta_{1}X_{bl}$ |
| Late unexplained residual model: regression analysis of residual of……. expected time use during and after lockdown to outcome measure^a^ | $Y=\alpha_{2}+\gamma_{2}X_{resdl}$  $Y=\alpha_{3}+\gamma_{3}X_{resal}$ |
| Conditional regression model, adding the residual of the expected……. time use during and after lockdown to early model | $Y=\alpha_{4}+\beta_{4}X_{bl}+\gamma_{2}X_{resdl}+\gamma_{3}X_{resal}$ |

Variables: $X_{bl}$, expected time use before lockdown ; $X_{resdl}$, residual of expected time use during lockdown, based on time use before lockdown; $X_{resal}$, residual of expected time use after lockdown, based on time use before lockdown; Y, expected outcome; $\propto$, intercept; $\beta$ and $\gamma$, coefficients.

^a^ First, time use during $X_{edl}$ is calculated, based on time use before lockdown $\alpha_{0}+\beta_{0}X_{bl}$. Then, the residual for expected time use during lockdown is calculated as $X_{resdl}=X_{dl}-X_{edl}$

^b^ second, time use after$X_{eal}$ is calculated, based on time use before and during lockdown $\alpha_{0}+\beta_{0}X_{bl}+\beta_{1}X_{dl}$. Then, the residual for expected time use after lockdown is calculated $X_{resal}=X_{al}-X_{eal}$

​

**A**

**B**

***

***

Supplementary figure 1: Time spent outdoors (A) and on near work (B) at age 13 in hours/day in spring, summer, winter and autumn. Difference compared to Spring : ***=P<0.001
